# A Novel and Convenient Method for Early Warning of Algal Cell Density by Chlorophyll Fluorescence Parameters and Its Application in a Highland Lake

**DOI:** 10.3389/fpls.2018.00869

**Published:** 2018-06-28

**Authors:** Huan Wang, Rong Zhu, Jia Zhang, Leyi Ni, Hong Shen, Ping Xie

**Affiliations:** ^1^Donghu Experimental Station of Lake Ecosystems, State Key Laboratory of Freshwater Ecology and Biotechnology of China, Institute of Hydrobiology, Chinese Academy of Sciences, Wuhan, China; ^2^University of Chinese Academy of Sciences, Beijing, China; ^3^State Key Laboratory of Plateau Ecology and Agriculture, Qinghai University, Xining, China

**Keywords:** chlorophyll fluorescence, *Microcystis* bloom, generalized linear mixed models, trigonometric regression, Phyto-PAM, algal density, the time lag

## Abstract

The occurrence of algal blooms in drinking water sources and recreational water bodies have been increasing and causing severe environmental problems worldwide, particularly when blooms dominated by *Microcystis* spp. Bloom prediction and early warning mechanisms are becoming increasingly important for preventing harmful algal blooms in freshwater ecosystems. Chlorophyll fluorescence parameters (CFpars) have been widely used to evaluate growth scope and photosynthetic efficiency of phytoplankton. According to our 2-year monthly monitor datasets in Lake Erhai, a simple but convenient method was established to predict *Microcystis* blooms and algal cell densities based on a CFpar representing maximal photochemical quantum yield of Photosystems II (PSII) of algae. Generalized linear mixed models, used to identify the key factors related to the phytoplankton biomass in Lake Erhai, showed significant correlations between Chl *a* concentration and both the light attenuation coefficient and water temperature. We fitted seasonal trends of CFpars (*F*_v_/*F*_m_ and Δ*F*/*F*_m_′) and algal cell densities into the trigonometric regression to predict their seasonal variations and the autocorrelation function was applied to calculate the time lag between them. We found that the time lag only existed between *F*_v_/*F*_m_ from blue channel and algal cell densities even both *F*_v_/*F*_m_ and Δ*F*/*F*_m_′ show the significant non-linear dynamics relationships with algal cell densities. The peak values of total algal cell density, cyanobacteria density and *Microcystis* density followed the foregoing peak value of *F*_v_/*F*_m_ from blue channel with a time lagged around 40 days. Therefore, we could predict the possibilities of *Microcystis* bloom and estimate the algal cell densities in Lake Erhai ahead of 40 days based on the trends of *F*_v_/*F*_m_ values from blue channel. The results from our study implies that the corresponding critical thresholds between *F*_v_/*F*_m_ value and bloom occurrence, which might give new insight into prediction of cyanobacteria blooms and provide a convenient and efficient way for establishment of early warning of cyanobacteria bloom in eutrophic aquatic ecosystems.

## Introduction

Harmful algal blooms (HABs) in freshwater ecosystems are subject of serious concern for ecosystems and human health because they reduce the quality and quantity of habitat for plants and animals, disrupt food web dynamics, create hypoxic zones, and produce toxins (Paerl et al., 2001; Miller et al., 2017). Changing environmental conditions like drought, increased water temperature and low water levels (Paerl et al., 2001; de Figueiredo et al., 2004; Qin et al., 2010; Watson et al., 2017), can increase the intensity and frequency of algal blooms. Further, through its effects on regional and local climatic patterns, climate change is also modifying patterns of HAB (Michalak et al., 2013). Therefore, predicting the HABs has become increasingly important for environmental and public health management.

Many efforts and resources have been devoted to forecasting algal blooms using mathematical modeling through quantitive indicators and environmental drivers. One of the most extensively applied models of predicting blooms are the parametric models (Wong et al., 2007; Gill et al., 2017). For example, the Baltic Operational Oceanographic System (BOOS) is a real-time oceanic observation system combining ecological forecast models for algal bloom in Baltic sea with annual water forecasts for the Baltic sea^1^. Artificial neural networks (ANNs) provide an alternative to parametric forecast models, where several environmental factors act as input variables to estimate the evolution of algal bloom and predict cell densities of freshwater phytoplankton species (Recknagel et al., 1997; Lee et al., 2003; Muttil and Chau, 2006). Statistical methods such as cross-correlation (Trimbee and Prepas, 1987), and generalized additive model (Lamon et al., 1996; Tao et al., 2012), as well as the development of satellite remote sensing forecasting techniques (Stumpf, 2001; Kutser, 2004), are also other possible options for predicting the occurrence of HAB. Despite having good predictive accuracy, all these methods have the major drawback of being time and labor consuming as well as complex in their calculation. Accuracy of these methods also rely on selecting a suitable set of parameters and models according to different lake conditions, nutrient status, and different local meteorological and hydrological conditions. Hence, previous studies have highlighted the need for simple, rapid, and geographically non-restricted approaches to predict algae blooms.

All the methods mentioned above are based on the relationships between algal growth and environmental factors, but rarely use physiological parameters of algae for bloom prediction. Chlorophyll fluorescence parameters (CFpars), *F*_v_/*F*_m_ and Δ*F*/*F*_m_′, can be considered as the main indicators for assessment of the photosystem II efficiency and for the photosynthetic capacity of algae (Misra et al., 2012). The fluorescence ratio *F*_v_/*F*_m_ refers to the photosynthetic activity and is taken as an algae viability assessment. Similarly,Δ*F*/*F*_m_′ reflects the actual physiological activity of PS II (Genty et al., 1990). Previous studies have shown that both *F*_v_/*F*_m_ and Δ*F*/*F*_m_′ respond to changes of environmental factors such as nutrients and light intensity and are directly related to the growth of algae (Boyd et al., 1999; Misra et al., 2012; Shi et al., 2016). Therefore, the use of these CFpars may be suitable candidates for simple predictions of algal blooms. To this end, pulse amplitude modulated (PAM) fluorometry is a promising analytical technique that measures the photochemical efficiency of photosystem II in phytoplankton; one of the most common, non-invasive and rapid existing indicators of the viability condition of phytoplankton in a sample irrespective of their size (Schreiber et al., 1995b; White et al., 2011; Kalaji et al., 2014, 2017). Furthermore, Phyto-PAM fluorometry procedure can distinguish the ratios of fluorescence yields of cyanobacteria, green algae and diatoms/dinoflagellates and output as different channels (blue channel, green channel and brown channel, respectively) (Dorigo and Leboulanger, 2001; Schmitt-Jansen and Altenburger, 2008). In the case of cyanobacteria (blue channel), almost no Chl fluorescence is excited by blue light (470 nm), while excitation at 645 nm is particularly strong due to phycocyanin and allophycocyanin absorption.

Highland lakes are distinctive unique ecosystems because they are subjected to extreme environmental conditions, such as strong radiation, low water temperature, relatively low nutrient conditions, and relatively simple food webs with low species abundance (Tolotti et al., 2006). As a result, these lakes have low buffering capacity and are very sensitive to climate change and other anthropogenic influences (Psenner and Schmidt, 1992; Psenner, 2002). Therefore, this sensitivity and responsiveness of the phytoplankton community in plateau lakes to external environmental stress makes them an ideal system for the purpose of this study. Highland lakes are also increasingly exposed to human activity globally. Common impacts include wastewater discharge from farmlands and households, fish introduction, transport and tourism pollution. These impacts are generating increased eutrophication, disappearance of aquatic vegetation, and algae blooms highland aquatic ecosystems (Tolotti et al., 2006; Huang et al., 2014). Notwithstanding these unfolding environmental problems, few studies have examined bloom forecast in highland lakes.

Lake Erhai, a typical high altitude lake in the Chinese Yunnan Province, has suffered increasingly frequent cyanobacterial blooms despite the relative lower nutrients and higher illumination characteristic of highland lakes compared to lowland lakes (Xu, 1996; Paerl et al., 2011). These responses are difficult to model explicitly according to conventional models based on quantitive indicators and environmental drivers. Before 1970s, Lake Erhai was an oligotrophic lake (Jin et al., 2005). Since the 80s, however, the lake has been affected by man-made eutrophication resulting from the growing resident population (Jin et al., 2005). This situation exacerbated after the 90s, as district population and human activities continued to increase, resulting in frequent cyanobacterial blooms (Wu and Wang, 1999). Large scale *Anabaena*-dominated cyanobacterial bloom firstly appeared during the summer of 1996 (Dong, 1999). However, the dominant cyanobacterial species during summer shifted to *Microcystis* after 2008 (Wen and Ma, 2011; Wei et al., 2012), coincident with an increase in bloom frequency and intensity. Here, we explore the potential for using multiwavelength Phyto-PAM fluorometry as a simple early warning forecast method for *Microcystis* blooms based on field data collected monthly over 2 years, with a focus on predictive performance and methodological constraints. Our research should give new insight into prediction of cyanobacteria blooms and provide a convenient and efficient way for the establishment of early warning systems of cyanobacterial blooms in eutrophic aquatic ecosystems.

## Materials and Methods

### Study Site and Sampling Method

**FIGURE 1:**
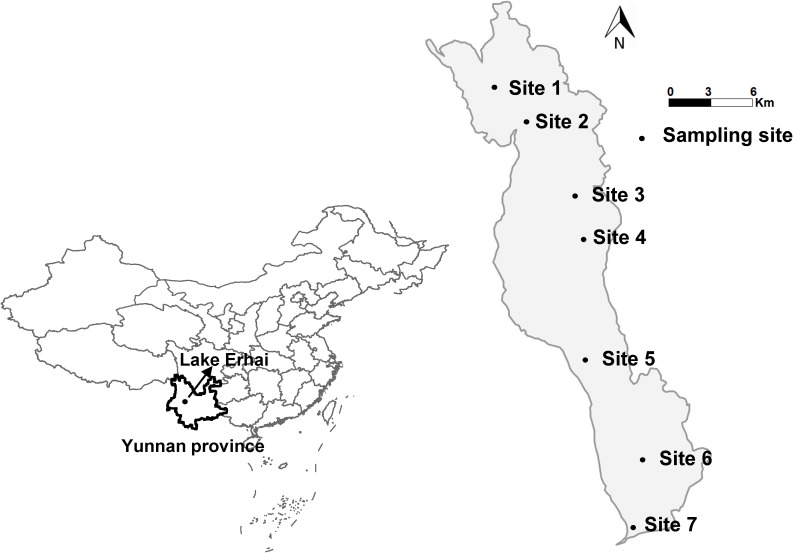
A map of Lake Erhai with the location of the sampling sites.

Data presented in this study correspond to a 2-year (June 2013–May 2015) field survey conducted in Lake Erhai (25°36′–25°58′ N, 100°05′–100°17′ E), the second largest high-altitude freshwater lake of the Yunnan Highlands in China with the normal elevation is 1974 m, to trace algal dynamics and *Microcystis* bloom. Water samples were taken monthly from three water depths (surface, middle, and bottom) at seven sites (**Figure 1**), then pooled for the measurement of physicochemical parameters, physiological indicators, and algal densities at each site. The water samples were stored in transparent glass bottles of 2.5 L and kept bottles half full. After sampling was completed (within 5 h), we measured the CFpars and physicochemical parameters immediately in the laboratory.

### Identification of Phytoplankton

One-L water samples were immobilized by 1% Lugol’s iodine solution and concentrated to 50 ml by a siphon after sedimentation for 48 h in Utermhol chambers to analyze the phytoplankton composition (Huang et al., 1999). Concentrated samples (0.1 ml) were thereafter counted and measured under 400× magnification using an Olympus microscope (Olympus BX21, Tokyo, Japan) after mixing. Colonial *Microcystis* cells were separated using an ultrasonic device (JY88-II, Scientiz, Ningbo, Zhejiang, China) and their constituent cells counted. Taxonomic identification of the phytoplankton species was performed according to Hu and Wei (Hu, 2006).

### Measurements of Physicochemical Parameters

All samples for nutrient and chlorophyll *a* determination were stored in the portable refrigerator (0°C) in the field and analyzed immediately upon returning to the laboratory. Samples for total phosphorus (TP), dissolved total phosphorus (DTP), dissolved inorganic phosphorus (DIP), total nitrogen (TN), nitrate (${NO}_{3}^{-}$), ammonium (${NO}_{4}^{+}$), and chlorophyll *a* (Chl *a*) concentrations were analyzed following standard preservation and analytical procedures of the Water Environment Federation (Association et al., 1915). The concentrations of Chl *a* was determined by spectroradiometer (SHIMADZU UV-2550, Japan) after appropriate aliquots (200–1000 ml) were filtered through Whatman GF-C glass microfiber filters and 24 h extraction in 90% acetone at 4°C in the dark. The absorbance of the processed samples was recorded at two different wavelengths (665 and 750 nm) following the protocol of Lorenzen (1967) for calculating Chl *a* concentration. Water temperature (T), pH value, dissolved oxygen (DO), and conductivity (COND) were measured onsite at 0.5 m below the water surface with a YSI ProPlus multiparameter water quality meter (Yellow Springs, OH, United States). The Secchi depth (SD) was assessed with a black and white Secchi disk (20 cm in diameter) to determine water transparency. PAR was measured at water depths of 0, 0.5, 1.0, 1.5, and 2.0 m using an underwater radiation sensor (UWQ-8342) connected to a data logger (Li-1400; Li-Cor Company, Lincoln, NE, United States). Light attenuation coefficient of water column (K) was calculated based on the equation: I_d_ = I_s_(1-K)/K, where I_d_ and I_s_ are irradiance at the corresponding water depth and water surface, respectively (Duarte et al., 1986).

### Measurements Chlorophyll Fluorescence Parameters

A Phyto-PAM (PHYTO-PAM Phytoplankton Analyzer, Heinz Walz GMBH, Effeltrich, Germany) was used to measure the maximum quantum yield [*F*_v_/*F*_m_ = (*F*_m_ – *F*_0_)/*F*_m_] and the effective quantum yield [Δ*F*/*F*_m_′ = (*F*_m_′ – *F*_0_′)/*F*_m_′] (Kühl et al., 2001). PAM fluorometry sensors estimate photosynthetic activity by comparing fluorescence yield of PSII under ambient irradiance (*F*) and after application of a saturating pulse (*F*_m_) (Bilger et al., 1995; Schreiber et al., 1995a; Schreiber, 2004). *F*_0_ and *F*_m_ are the minimum and maximum fluorescence of a dark-adapted sample during a saturating light pulse, respectively. Similarly, *F*_0_′ and *F*_m_′ are the minimum and maximum fluorescence of a light-adapted sample during a saturating light pulse. Because emission wavelengths (peaking at 470, 520, 645, and 665 nm) do not correspond to the peak wavelengths of absorption of the relevant pigments, the deconvolution procedure requires the ratios of fluorescence yields of cyanobacteria, green algae, and diatoms/dinoflagellates to show pronounced differences upon excitation with these wavelengths.

### Statistics and Inferences

**FIGURE 2:**
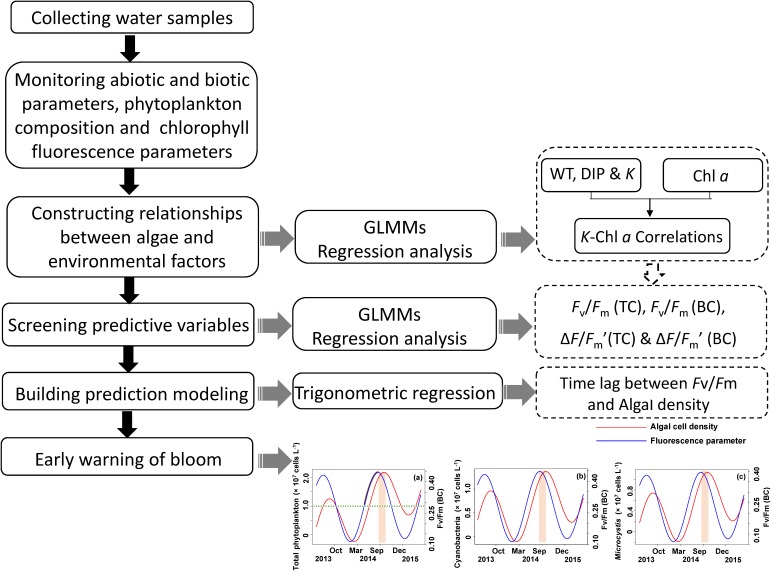
Technique flow diagram of the application of method early warning the algal cell density and *Microcystis* bloom by measuring chlorophyll fluorescence *F*_v_/*F*_m_ (BC).

The analytical process is schematically shown in **Figure 2**. Generalized linear mixed models (GLMMs) (Bolker et al., 2009) were used to detect the key environment drivers (i.e., light, temperature, and nutrient) correlated to phytoplankton biomass (Chl *a*) during the period of field monitoring. In present study, we used Chl *a* as a measure of algal biomass according to previous studies in both freshwater and marine ecosystems (Carlson, 1977; Barlow et al., 1993; Schlüter et al., 2000; Chen et al., 2003). Sampling site within the lake was introduced as a random effect in the model to avoid pseudoreplication by introducing correlation among species (Hurlbert, 1984). The random effects might also account for some unknown factors that influence the phytoplankton biomass in the lake, such as differences in flow velocity and nutrient concentration among sampling sites. Variables of environment drivers were transformed using square root to normalize the data for analysis.

Generalized linear mixed models (Bolker et al., 2009) was also used to test whether CFpars can predict algae density or biomass. The total phytoplankton cell density (*C_t_*), cyanobacteria cell density (*C_c_*), and *Microcystis* cell density (*C*_m_) were used as response variables. The *F*_v_/*F*_m_ from total channel [*F*_v_/*F*_m_ (TC)], *F*_v_/*F*_m_ from blue channel [*F*_v_/*F*_m_ (BC)], Δ*F*/*F*_m_′ from total channel [Δ*F*/*F*_m_′ (TC)] and Δ*F*/*F*_m_′ from blue channel [Δ*F*/*F*_m_′ (BC)] were used as predictor variables. Site effects were also incorporated as random effect in these models.

Informed by the results from the GLMMs, we built a bloom prediction model by fitting a seasonal trigonometric regression to each cell density parameter (*C_t_*, *C_c_*, and *C*_m_) and the values of *F*_v_/*F*_m_ and Δ*F*/*F*_m_′ according to the following equation (Pollock, 2000):

$$y=\beta_{0}+\beta_{1}x+\beta_{2}\sin(2\pi x)+\beta_{3}\cos(2\pi x)+\epsilon$$

Where *y* is cell densities (*C_t_*, *C_c_*, or *C*_m_) or *F*_v_/*F*_m_ or Δ*F*/*F*_m_′, x is time (month), β_0_ is the intercept and β_1_ is the slope of the regression, which represent stochastic local trend components; β_2_ and β_3_ are the coefficients of the trigonometric (cyclical) seasonal components [sin*(2πx)* and cos*(2πx)*]. The error term is represented by ε. The values of *x* and *y* were selected at random for running the trigonometric regressions and Each model ran 9999 times for re-randomization tests and the cross correlations between cell density and CFpars were calculated by the autocorrelation function (ACF) with associated confidence intervals at the 0.05 level. Cross-correlation values can be considered as the time lag between cell density and CFpars, which are reported as mean and standard deviation.

All statistical analyses were conducted in R 3.1.0 (R Core Team, 2014) using the packages reshape2 (Wickham, 2007), lme4 (Bates et al., 2011), and ggplot2 (Wickham, 2009).

## Results

### Phytoplankton Cell Densities

**FIGURE 3:**
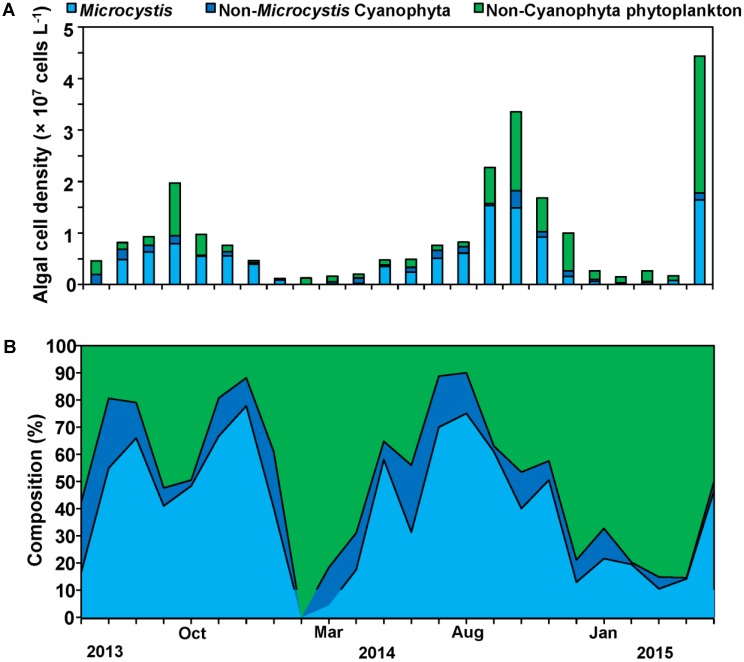
Cell density successions **(A)** and composition successions **(B)** of *Microcystis*, Non- *Microcystis* Cyanophyta and Non-Cyanophyta phytoplankton cell density.

Our sampling campaign lasted 2 years and included two cyanobacteria bloom phases. Cyanophyta was the major phylum of phytoplankton during the whole year with 50% of total phytoplankton cell density, and *Microcystis* was the overwhelming dominant genus during the periods of cyanobacterial blooms with 78% of total cyanobacterial cell density (**Figure 3**). At specific bloom phases *Microcystis* reached up to 80% of all cyanobacterial cell density with the cell densities exceeding 1 × 10^7^ cells L^-1^, while those of cyanobacterial exceeded 1.5 × 10^7^ cells L^-1^ comprising 60% of all phytoplankton cell density (**Figures 3A,B**).

### Driving Factors of Phytoplankton Cell Densities

**FIGURE 4:**
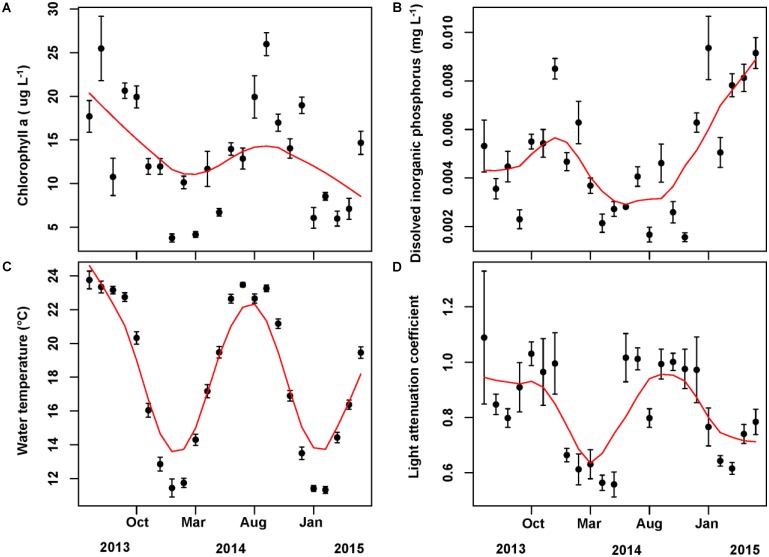
Monthly time series of Chlorophyll *a* **(A)**, dissolved inorganic phosphorus **(B)**, water temperature **(C)**, and light attenuation coefficient **(D)**. The mean value and associated standard deviation among sites is shown in each panel.

**FIGURE 5:**
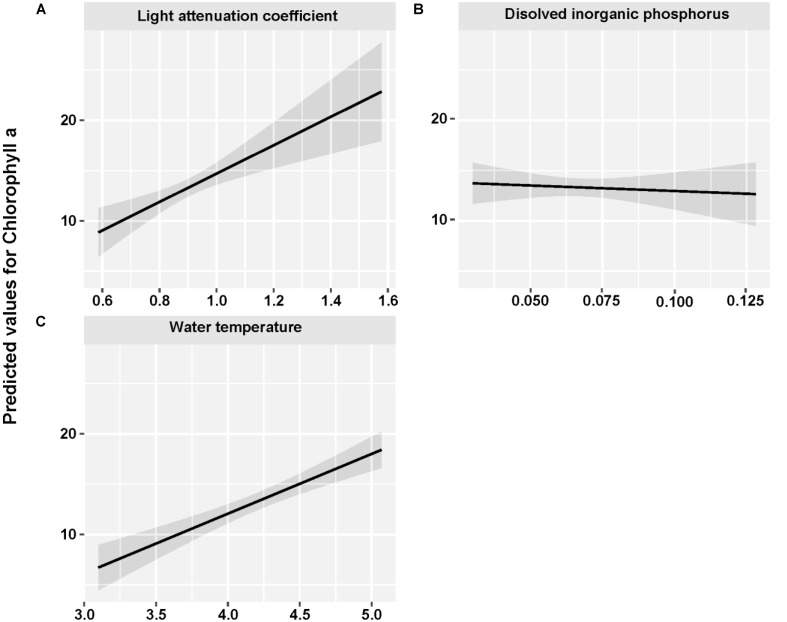
Fitted light attenuation coefficient **(A)**, dissolved inorganic phosphorus **(B)**, and water temperature **(C)** for Chlorophyll *a* by using GLMMs analysis.

The mean concentration of Chl *a* during the sampling period (June 2013 to May 2015) was 13.33 ug/L, with a peak value exceeding 30 ug/L. Water temperature and light attenuation coefficient followed similar seasonal variations (**Figure 4**). The maximum level of water temperature encountered in Lake Erhai was 25.7°C, and the mean water temperature during sampling period was of 18.0°C (**Figure 4C**). Light attenuation coefficient showed a mean of 0.83 and a maximum of 2.49 (**Figure 4D**). The results from GLMMs model showed a highly significant relationship (*p* < 0.0001) of both water temperature and light attenuation coefficient to Chl *a* concentration (**Figure 5**). In contrast, all nutrient parameters, pH, DO, SD, and COND showed lower non-significant correlations with Chl *a* concentration (data not shown). The annual change of water temperature and the light attenuation coefficient in Lake Erhai, characteristic of a highland lake, and their close relationship to Chl *a*, suggest a potential relationship between algal cell densities, photosynthetic activity and the seasonal succession of algae, which can be predicted directly/indirectly by measuring the fluorescence parameters.

### Testing Parameters of GLMMs

To test the practicability of using the CFpars for determining the algal cell density in water columns, we used *F*_v_/*F*_m_ (TC), *F*_v_/*F*_m_ (BC), Δ*F*/*F*_m_′ (TC), Δ*F*/*F*_m_′ (BC), *C_t_*, *C_c_*, and *C*_m_ fitting time cycle changes of GLMMs. All parameters were significantly correlated with seasonality (*p* < 0.05). Further, *F*_v_/*F*_m_ (TC), *F*_v_/*F*_m_ (BC), and *C*_m_ were highly significantly correlated with seasonal variation (*p* < 0.001) (**Table 1**). As a result, all these parameters could be potentially selected for model creation to estimate phytoplankton cell density by fluorescence, where model prediction of algal cell density is a function of its Chlorophyll light response.

**Table 1:** Values (mean ± SD, ranges in parentheses) of *F*_v_/*F*_m_ (TC), *F*_v_/*F*_m_ (BC), Δ*F*/*F*_m_′ (TC), Δ*F*/*F*_m_′ (BC), *C_t_*, *C*_c,_ and *C*_m_ using GLMMs with two stochastic (intercept β_0_ and slope β_1_) local trend components and two trigonometric (cyclical) seasonal components (sin β_2_ and cos β_3_ pairs).

	β_0_	β_1_	β_2_	β_3_	Model adj R^2^	Model F	Model *p*-value
*F*_v_/*F*_m_ (TC)	0.53 ± 0.01 (*p* < 0.001)	-0.00 ± 0.00 (*p* < 0.05)	0.04 ± 0.01 (*p* < 0.001)	-0.01 ± 0.01 (ns)	0.71	*F*_3,20_ = 19.92	*p* < 0.001
*F*_v_/*F*_m_ (BC)	0.27 ± 0.08 (*p* < 0.01)	0.00 ± 0.01 (ns)	0.16 ± 0.05 (*p* < 0.01)	0.04 ± 0.05 (ns)	0.25	*F*_3,20_ = 3.56	*p* < 0.05
Δ*F*/*F*_m_′ (TC)	0.34 ± 0.023 (*p* < 0.001)	0.00 ± 0.00 (ns)	0.07 ± 0.02 (*p* < 0.001)	-0.01 ± 0.01 (ns)	0.47	*F*_3,20_ = 7.75	*p* < 0.05
Δ*F*/*F*_m_′ (BC)	0.15 ± 0.06 (*p* < 0.05)	0.00 ± 0.00 (*p* < 0.05)	0.16 ± 0.04 (*p* < 0.01)	0.00 ± 0.04 (ns)	0.34	*F*_3,20_ = 4.9	*p* < 0.05
*C_t_*	22742 ± 4005336 (ns)	770943 ± 285584 (*p* < 0.05)	9190585 ± 2781086 (*p* < 0.01)	-2975061 ± 2584577 (ns)	0.32	*F*_3,20_ = 4.69	*p* < 0.05
*C_c_*	1360317 ± 1656293 (ns)	336873 ± 118095 (*p* < 0.01)	5917219 ± 1150040 (*p* < 0.001)	-2095533 ± 1068779 (ns)	0.54	*F*_3,20_ = 10.04	*p* < 0.001
*C*_m_	541413 ± 1564123 (ns)	330612 ± 111523 (*p* < 0.01)	5211725 ± 1086041 (*p* < 0.001)	-2068081 ± 1009302 (ns)	0.52	*F*_3,20_ = 9.17	*p* < 0.001


### Time Lag Between Algal Cell Density and Fluorescence Parameters

**FIGURE 6:**
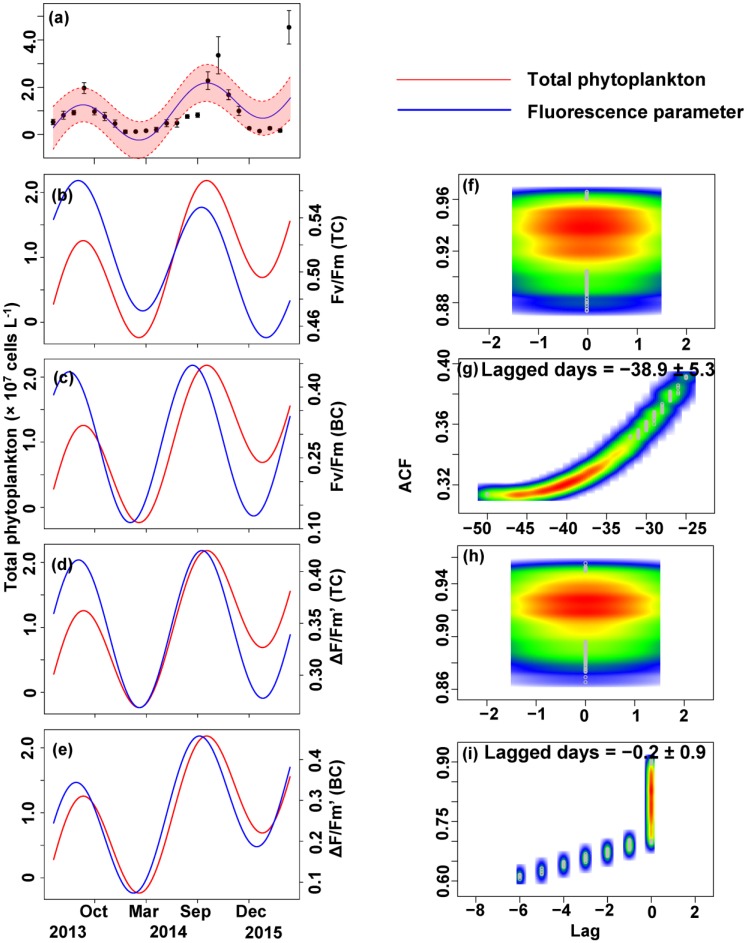
Trigonometric regression of the total phytoplankton cell density ( × 10^7^ cells L^-1^) **(a)** and fluorescence measurements [*F*_v_/*F*_m_ (TC) **(b)**, *F*_v_/*F*_m_ (BC) **(c)**, Δ*F*/*F*_m_′ (TC) **(d),** and Δ*F*/*F*_m_′ (BC) **(e)**]. We fitted these seasonal trends with a time-lag analysis [*F*_v_/*F*_m_ (TC) **(f)**, *F*_v_/*F*_m_ (BC) **(g)**, Δ*F*/*F*_m_′ (TC) **(h)**, and Δ*F*/*F*_m_′ (BC) **(i)**]. The legged days and associated standard deviation among sites is shown in each panel.

**FIGURE 7:**
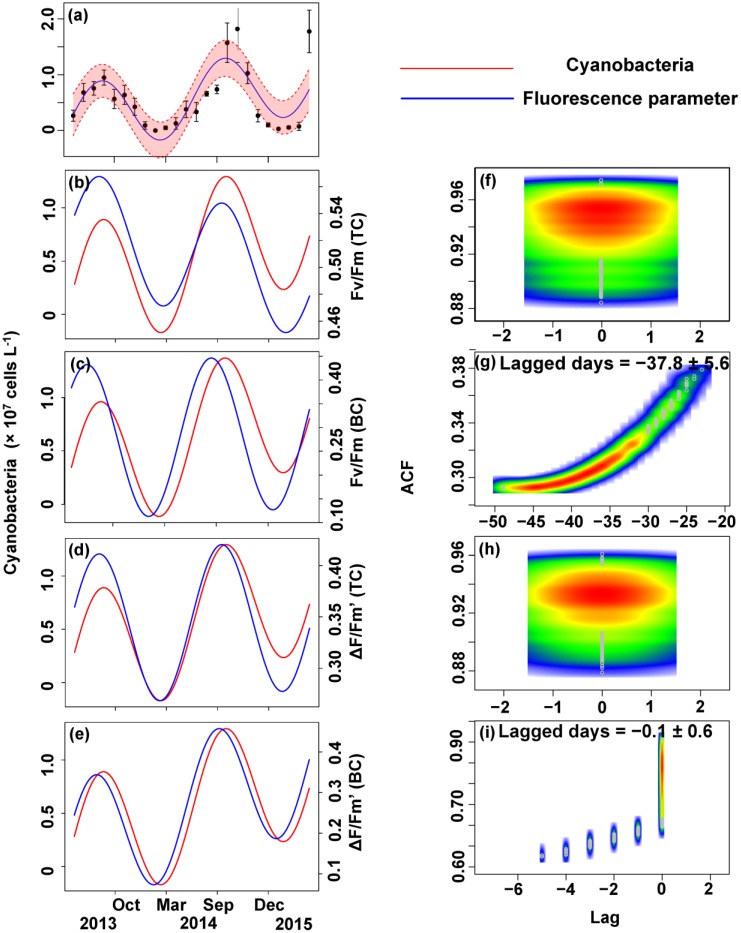
Trigonometric regression of the cyanobacteria cell density **(a)** and fluorescence measurements [*F*_v_/*F*_m_ (TC) **(b)**, *F*_v_/*F*_m_ (BC) **(c)**, Δ*F*/*F*_m_′ (TC) **(d)**, and Δ*F*/*F*_m_′ (BC) **(e)**]. We fitted these seasonal trends with a time-lag analysis [*F*_v_/*F*_m_ (TC) **(f)**, *F*_v_/*F*_m_ (BC) **(g)**, Δ*F*/*F*_m_′ (TC) **(h)**, and Δ*F*/*F*_m_′ (BC) **(i)**]. The legged days and associated standard deviation among sites is shown in each panel.

**FIGURE 8:**
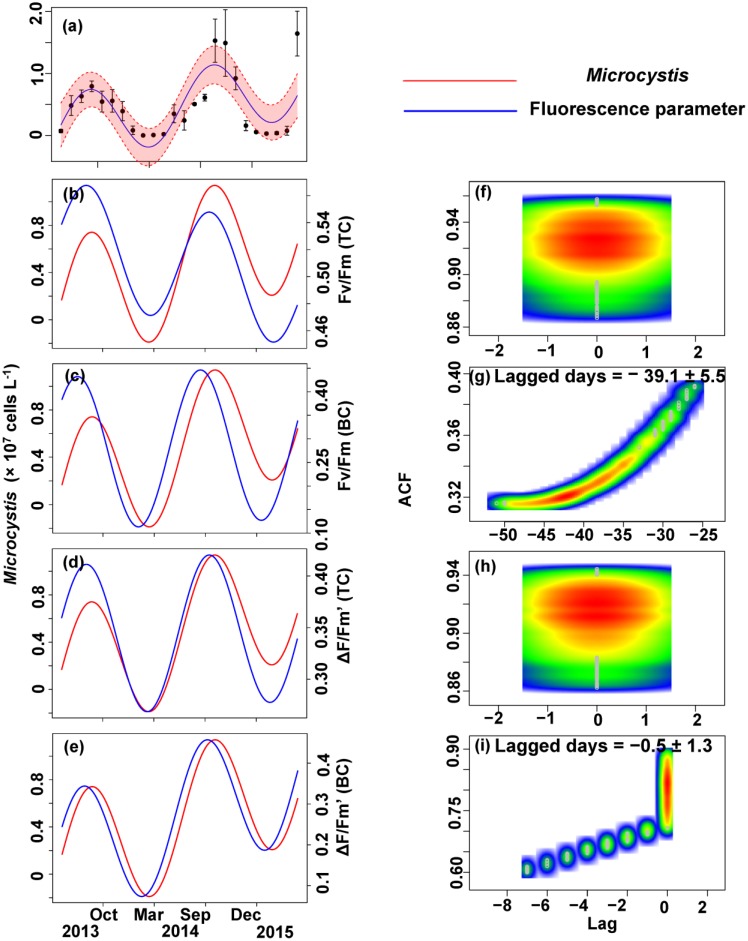
Trigonometric regression of the *Microcystis* cell density **(a)** and fluorescence measurements [*F*_v_/*F*_m_ (TC) **(b)**, *F*_v_/*F*_m_ (BC) **(c)**, Δ*F*/*F*_m_′ (TC) **(d)**, and Δ*F*/*F*_m_′ (BC) **(e)**]. We fitted these seasonal trends with a time-lag analysis [*F*_v_/*F*_m_ (TC) **(f)**, *F*_v_/*F*_m_ (BC) **(g)**, Δ*F*/*F*_m_′ (TC) **(h)**, and Δ*F*/*F*_m_′ (BC) **(i)**]. The legged days and associated standard deviation among sites is shown in each panel.

The relationship between the algal cell density and *F*_v_/*F*_m_ or Δ*F*/*F*_m_′ value was also first identified by GLMMs, then fitted using trigonometric regression. We found significant positive non-linear correlations between fluorescence parameters and cell density (**Figures 6**–**8**).

No apparent time lag was found between *F*_v_/*F*_m_ (TC) and *C_t_* (**Figures 6b,f**) or *C_c_* (**Figures 7b,f**) or *C*_m_ (**Figures 8b,f**). However, the time lag between *F*_v_/*F*_m_ (BC) and *C_t_* (**Figures 6b,f**) or *C_c_* (**Figures 7b,f**) or *C*_m_ (**Figures 8b,f**) was almost 40 days. *F*_v_/*F*_m_ (BC) lead on average *C_t_* by 38.9 ± 5.3 days (**Figures 4C,D**). *F*_v_/*F*_m_ (BC) lead *C_c_* by 37.8 ± 5.6 days (**Figures 7c,g**). *F*_v_/*F*_m_ (BC) forward lead *C*_m_ by 39.1 ± 5.5 (**Figures 8c,g**). Similarly, no time lag was found betweenΔ*F*/*F*_m_′ (TC) and neither *C_t_* (**Figures 6d,h**), C_c_ (**Figures 7d,h**), or C_m_ (**Figures 8d,h**). Time lags were found in the other parameters, with the Δ*F*/*F*_m_′ (BC) leading total phytoplankton cell density by 0.2 ± 0.9 days (**Figures 6e,i**); Δ*F*/*F*_m_′ (BC) leading *C_c_* by 0.1 ± 0.6 days (**Figures 7e,i**); andΔ*F*/*F*_m_′ (BC) leading *C*_m_ by 0.5 ± 1.3 days (**Figures 8e,i**).

### Application of F_v_/F_m_ (BC) to Early Warning of *Microcystis* Blooms

**FIGURE 9:**
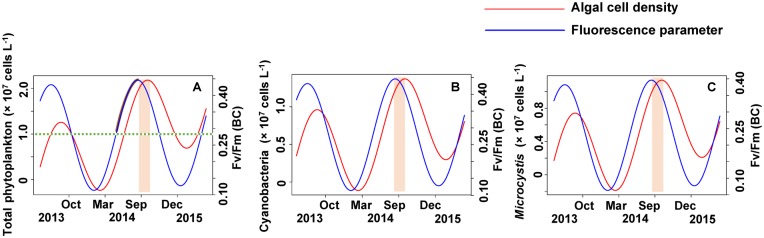
The early warning of algal cell density and *Microcystis* bloom by simulating the time lag between *F*_v_/*F*_m_ (BC) and algal cell density. **(A)** Total phytoplankton, **(B)** Cyanobacteria, **(C)** *Microcystis*.

According to the strong time lag between *F*_v_/*F*_m_ and cell density, forecasting *Microcystis* bloom and the cell density in Lake Erhai should be possible by in lake monitoring of the *F*_v_/*F*_m_ (BC) value (**Figure 9**). The trigonometric regression (**Figure 9A**) shows that the likelihood of a cyanobacteria bloom can increase when the value of *F*_v_/*F*_m_ reach 0.28 and the trend keeps upward. But if the trend decreases, the possibility of cyanobacteria bloom can become low even if the value of *F*_v_/*F*_m_ remains higher than 0.28. If the trend of *F*_v_/*F*_m_ declines and the *F*_v_/*F*_m_ value is lower than 0.28, a cyanobacteria bloom seems unlikely. Here, we define 10^7^ cells L^-1^ as the threshold value for a cyanobacteria bloom. The peak value of *F*_v_/*F*_m_ (BC) is usually followed by a peak value of phytoplankton after approximately 40 days.

## Discussion

In present study, we developed a novel method to predict *Microcystis* bloom via physiological parameters of algae and provided a rapid and simple way of early warning for blooms. Compare to the common approaches (direct physicochemical measurements combined with regular monitoring) of bloom forecast (Trimbee and Prepas, 1987; Recknagel et al., 1997; Wong et al., 2007; Winder and Sommer, 2012; Ruiz-de la Torre et al., 2013; Gill et al., 2017), our method could save both time and labor by handling just one forecast factor through a single operation. Compare to the remote sensing approaches, the effective ways which widely used for short-term bloom forecast by using satellite and airborne measurements of spectral reflectance of water color (Wynne et al., 2013; Gill et al., 2017) even sometimes they were limited to the use of cloudless remote-sensing images and constrained by pixels, our method emphasized on chlorophyll fluorescent parameters (*F*_v_/*F*_m_) instead of monitoring Chl *a* concentrations. A similar method for bloom prediction has not been reported so far. Both abiotic environmental factors (O’neil et al., 2012; Paerl and Paul, 2012; Winder and Sommer, 2012; Ruiz-de la Torre et al., 2013) and biotic factors (Provasoli, 1958; González et al., 2000; Chattopadhayay et al., 2002) could affect the concentrations of Chl *a* and trigger cyanobacterial blooms. Unlike the effects of environmental factors on phytoplankton, the maximum quantum yield (*F*_v_/*F*_m_) indicates directly photosynthetic activity of phytoplankton (Schreiber et al., 1995b; Shi et al., 2016). *F*_v_/*F*_m_ can drop significantly when algae in response to changing environmental conditions (Shen and Song, 2007; Shi et al., 2016). Therefore, *F*_v_/*F*_m_ can be considered as a sensitive indicator that can reflect algae viability assessment (Genty et al., 1990; Oxborough and Baker, 1997; Boyd et al., 2000).

The GLMMs, trigonometric regression and ACF are the main analytical models used in our method for prediction of time lag responses in dynamics of phytoplankton. GLMMs model are a popular and widely used method for selecting driving factors in fisheries research (Venables and Dichmont, 2004) and plant litter decomposition (Veen et al., 2015), and seemed to successfully determine the dominant factors in our study. The trigonometric regression can effectively reflect and forecast the time series changes and seasonal trends of electricity demand (Harvey and Koopman, 1993; Zhou et al., 2006), but has not been applied before to algal bloom forecasting. Again, our results show that it can fit to the purpose of reconstructing seasonal patterns of cell density, *F*_v_/*F*_m_ and Δ*F*/*F*_m_′. The combination of results from the GLMMs and trigonometric regressions allowed in turn for the estimation of time lags between algal cell density and the fluorescence parameter using the ACF, and the random error was examined by Permutation test. Given light intensity play an important role in the dynamics of phytoplankton of Lake Erhai, the fluorescence parameters could capture the physiological characteristics of algae. The model created by fluorescence parameters had considerable predictive capacity of early bloom warning.

Our method should be applicable to algal bloom forecasting in other eutrophic lakes, but it might be not suitable for lakes where the diversity of phytoplankton is too high. Due to the complex pigment composition of chloroplast, each species of algae has its own excitation and emission wavelength, resulting in species-specific channel in different water environments through the fluorescence method (Schreiber, 2004). In the present study, cyanophyta was the clear dominant phylum and *Microcystis* the overwhelming dominant genus of cyanophyta. Thus, the blue channel value of *F*_v_/*F*_m_ can reflect the PSII function of *Microcystis* and infer the possible cell density. The forecasting ability of a model for early warning of algal blooms depends also on the quantity of data and the frequency of sampling (Andersen and Bollerslev, 1998; Ghysels et al., 2006). Whether monthly or higher sampling frequencies (e.g., fortnightly, weekly, or daily) are most appropriate for early warning by time lag analysis deserves further research. At the same time, an *in situ* measurement might be more helpful for accurate prediction.

## Conclusion

We have established a rapid, simple and convenient novel method to estimate the algal cell density in a plateau lake by measuring chlorophyll fluorescence *F*_v_/*F*_m_; a sensitive physiological parameter which directly reflects growth potentiality of algal and forecasts algal further growth rather than early warning of contamination. The traits of *F*_v_/*F*_m_ make it more efficient for prediction of algal bloom than using physicochemical parameters. Our study implies that in addition to the parameters of chlorophyll fluorescence, other physiological parameters of algal might also can be applied to the prediction of algal bloom. These results suggest using critical thresholds between *F*_v_/*F*_m_ value and bloom occurrence might give new insight into prediction of cyanobacteria blooms and provide a convenient and efficient way for establishment of early warning of cyanobacteria bloom in eutrophic aquatic ecosystems.

## Author Contributions

PX and HS designed the study. HW and RZ conducted the experiments. HW analyzed the data and led the manuscript writing. JZ and LN helped perform the analysis. HS revised the manuscript. All authors contributed to the final draft.

## Conflict of Interest Statement

The authors declare that the research was conducted in the absence of any commercial or financial relationships that could be construed as a potential conflict of interest.

## Footnotes

1 www.boos.org

## References

[B1] Andersen T. G. Bollerslev T. (1998). Answering the skeptics: yes, standard volatility models do provide accurate forecasts. *Int. Econ. Rev.* 394 885–905. 10.2307/2527343

[B2] Association A. P. H. Association A. W. W. Federation W. P. C. Federation W. E. (1915). *Standard Methods for the Examination of Water and Wastewater.* Washington, DC: American Public Health Association.

[B3] Barlow R. Mantoura R. Gough M. Fileman T. (1993). Pigment signatures of the phytoplankton composition in the northeastern Atlantic during the 1990 spring bloom. Deep Sea Research Part II. *Top. Stud. Oceanogr.* 40 459–477. 10.1016/0967-0645(93)90027-K

[B4] Bates D. Maechler M. Bolker B. (2011). *lme4: Linear Mixed-Effects Models using S4 Classes.* Available at: http://lme4.r-forge.r-project.org/

[B5] Bilger W. Schreiber U. Bock M. (1995). Determination of the quantum efficiency of photosystem II and of non-photochemical quenching of chlorophyll fluorescence in the field. *Oecologia* 102 425–432. 10.1007/BF00341354 28306885

[B6] Bolker B. M. Brooks M. E. Clark C. J. Geange S. W. Poulsen J. R. (2009). Generalized linear mixed models: a practical guide for ecology and evolution. *Trends Ecol. Evol.* 24 127–135. 10.1016/j.tree.2008.10.008 19185386

[B7] Boyd P. Laroche J. Gall M. Frew R. Mckay R. M. L. (1999). Role of iron, light, and silicate in controlling algal biomass in subantarctic waters SE of New Zealand. *J. Geophys. Res.* 104 13395–13408. 10.1029/1999JC900009

[B8] Boyd R. W. Watson A. J. Law C. S. Abraham E. R. (2000). A mesoscale phytoplankton bloom in the polar Southern Ocean stimulated by iron fertilization. *Nature* 407 695–702. 10.1038/35037500 11048709

[B9] Carlson R. E. (1977). A trophic state index for lakes. *Limnol. Oceanogr.* 22 361–369. 10.4319/lo.1977.22.2.0361

[B10] Chattopadhayay J. Sarkar R. Mandal S. (2002). Toxin-producing plankton may act as a biological control for planktonic blooms—field study and mathematical modelling. *J. Theor. Biol.* 215 333–344. 10.1006/jtbi.2001.2510 12054841

[B11] Chen Y. Fan C. Teubner K. Dokulil M. (2003). Changes of nutrients and phytoplankton chlorophyll-a in a large shallow lake, Taihu, China: an 8-year investigation. *Hydrobiologia* 506 273–279. 10.1023/B:HYDR.0000008604.09751.01

[B12] de Figueiredo D. R. Azeiteiro U. M. Esteves S. M. Gonçalves F. J. Pereira M. J. (2004). Microcystin-producing blooms—a serious global public health issue. *Ecotoxicol. Environ. Saf.* 59 151–163. 10.1016/j.ecoenv.2004.04.006 15327870

[B13] Dong Y. (1999). Research on blue algae plakton bloom in Erhai lake. *Yunnan Environ. Sci.* 4:010 10.13623/j.cnki.hkdk.1999.04.010

[B14] Dorigo U. Leboulanger C. (2001). A pulse-amplitude modulated fluorescence-based method for assessing the effects of photosystem II herbicides on freshwater periphyton. *J. Appl. Phycol.* 13 509–515. 10.1023/A:1012598816581

[B15] Duarte C. M. Kalff J. Peters R. H. (1986). Patterns in biomass and cover of aquatic macrophytes in lakes. *Can. J. Fish. Aquat. Sci.* 43 1900–1908. 10.1139/f86-235

[B16] Genty B. Harbinson J. Briantais J.-M. Baker N. R. (1990). The relationship between non-photochemical quenching of chlorophyll fluorescence and the rate of photosystem 2 photochemistry in leaves. *Photosynth. Res.* 25 249–257. 10.1007/BF00033166 24420355

[B17] Ghysels E. Santa-Clara P. Valkanov R. (2006). Predicting volatility: getting the most out of return data sampled at different frequencies. *J. Economet.* 131 59–95. 10.1016/j.jeconom.2005.01.004

[B18] Gill D. Ming T. Ouyang W. (2017). *Improving the Lake Erie HAB Tracker: A Forecasting & Decision Support Tool for Harmful Algal Blooms.* Available at: http://hdl.handle.net/2027.42/136562

[B19] González J. M. Simó R. Massana R. Covert J. S. Casamayor E. O. (2000). Bacterial community structure associated with a dimethylsulfoniopropionate-producing North Atlantic algal bloom. *Appl. Environ. Microbiol.* 66 4237–4246. 10.1128/AEM.66.10.4237-4246.2000 11010865 PMC92291

[B20] Harvey A. Koopman S. J. (1993). Forecasting hourly electricity demand using time-varying splines. *J. Am. Statist. Assoc.* 88 1228–1236. 10.2307/2291261

[B21] Hu H. (2006). *The Freshwater Algae of China: Systematics, Taxonomy and Ecology.* Ottawa, ON: Science Press.

[B22] Huang C. Wang X. Yang H. Li Y. Wang Y. (2014). Satellite data regarding the eutrophication response to human activities in the plateau lake Dianchi in China from 1974 to 2009. *Sci. Total Environ.* 485 1–11. 10.1016/j.scitotenv.2014.03.031 24698830

[B23] Huang X. Chen W. Cai Q. (1999). *Survey, Observation and Analysis of Lake Ecology.* Beijing: Science Press.

[B24] Hurlbert S. H. (1984). Pseudoreplication and the design of ecological field experiments. *Ecol. Monogr.* 54 187–211. 10.2307/1942661 15810678

[B25] Jin X. Xu Q. Huang C. (2005). Current status and future tendency of lake eutrophication in China. *Sci. China Ser. C* 48 948–954. 10.1360/062005-286 16512216

[B26] Kalaji H. M. Schansker G. Brestic M. Bussotti F. Calatayud A. (2017). Frequently asked questions about chlorophyll fluorescence, the sequel. *Photosynth. Res.* 132 13–66. 10.1007/s11120-016-0318-y 27815801 PMC5357263

[B27] Kalaji H. M. Schansker G. Ladle R. J. Goltsev V. Bosa K. (2014). Frequently asked questions about in vivo chlorophyll fluorescence: practical issues. *Photosynth. Res.* 122 121–158. 10.1007/s11120-014-0024-6 25119687 PMC4210649

[B28] Kühl M. Glud R. N. Borum J. Roberts R. Rysgaard S. (2001). Photosynthetic performance of surface-associated algae below sea ice as measured with a pulse amplitude-modulated (PAM) fluorometer and O2 microsensors. *Mar. Ecol. Prog. Ser.* 223 1–14. 10.3354/meps223001

[B29] Kutser T. (2004). Quantitative detection of chlorophyll in cyanobacterial blooms by satellite remote sensing. *Limnol. Oceanogr.* 49 2179–2189. 10.4319/lo.2004.49.6.2179

[B30] Lamon E. C. Reckhow K. H. Havens K. E. (1996). Using generalized additive models for prediction of chlorophyll *a* in Lake Okeechobee, Florida. *Lakes Reservoirs Res. Manag.* 2 37–46. 10.1111/j.1440-1770.1996.tb00046.x

[B31] Lee J. H. Huang Y. Dickman M. Jayawardena A. W. (2003). Neural network modelling of coastal algal blooms. *Ecol. Model.* 159 179–201. 10.1016/S0304-3800(02)00281-8

[B32] Lorenzen C. J. (1967). Determination of chlorophyll and pheo-pigments: spectrophotometric equations. *Limnol. Oceanogr.* 12 343–346. 10.4319/lo.1967.12.2.0343

[B33] Michalak A. M. Anderson E. J. Beletsky D. Boland S. Bosch N. S. (2013). Record-setting algal bloom in Lake Erie caused by agricultural and meteorological trends consistent with expected future conditions. *Proc. Natl. Acad. Sci. U.S.A.* 110 6448–6452. 10.1073/pnas.1216006110 23576718 PMC3631662

[B34] Miller T. R. Beversdorf L. J. Weirich C. A. Bartlett S. L. (2017). Cyanobacterial toxins of the laurentian great lakes, their toxicological effects, and numerical limits in drinking water. *Mar. Drugs* 15:E160. 10.3390/md15060160 28574457 PMC5484110

[B35] Misra A. N. Misra M. Singh R. (2012). *Chlorophyll Fluorescence in Plant Biology.* Available at: http://www.intechopen.com/books/biophysics/chlorophyll-fluorescence-in-plant-biology

[B36] Muttil N. Chau K.-W. (2006). Neural network and genetic programming for modelling coastal algal blooms. *Int. J. Environ. Pollut.* 28 223–238. 10.1504/IJEP.2006.011208

[B37] O’neil J. Davis T. Burford M. Gobler C. (2012). The rise of harmful cyanobacteria blooms: the potential roles of eutrophication and climate change. *Harm. Algae* 14 313–334. 10.1016/j.hal.2011.10.027

[B38] Oxborough K. Baker N. R. (1997). Resolving chlorophyll a fluorescence images of photosynthetic efficiency into photochemical and non-photochemical components–calculation of qP and Fv′/Fm′ without measuring Fo. *Photosynth. Res.* 54 135–142. 10.1023/A:1005936823310

[B39] Paerl H. W. Fulton R. S. Moisander P. H. Dyble J. (2001). Harmful freshwater algal blooms, with an emphasis on cyanobacteria. *Sci. World J.* 1 76–113. 10.1100/tsw.2001.16 12805693 PMC6083932

[B40] Paerl H. W. Paul V. J. (2012). Climate change: links to global expansion of harmful cyanobacteria. *Water Res.* 46 1349–1363. 10.1016/j.watres.2011.08.002 21893330

[B41] Paerl H. W. Xu H. Mccarthy M. J. Zhu G. Qin B. (2011). Controlling harmful cyanobacterial blooms in a hyper-eutrophic lake (Lake Taihu, China): the need for a dual nutrient (N & P) management strategy. *Water Res.* 45 1973–1983. 10.1016/j.watres.2010.09.018 20934736

[B42] Pollock D. (2000). Trend estimation and de-trending via rational square-wave filters. *J. Economet.* 99 317–334. 10.1016/S0304-4076(00)00028-2

[B43] Provasoli L. (1958). Nutrition and ecology of protozoa and algae. *Annu. Rev. Microbiol.* 12 279–308. 10.1146/annurev.mi.12.100158.001431 13595608

[B44] Psenner R. (2002). Alpine waters in the interplay of global change: complex links-simple effects. *Moun. Res. Dev.* 25 376–385.

[B45] Psenner R. Schmidt R. (1992). Climate-driven pH control of remote alpine lakes and effects of acid deposition. *Nature* 356:781 10.1038/356781a0

[B46] Qin B. Zhu G. Gao G. Zhang Y. Li W. (2010). A drinking water crisis in Lake Taihu, China: linkage to climatic variability and lake management. *Environ. Manag.* 45 105–112. 10.1007/s00267-009-9393-6 19915899

[B47] R Core Team (2014). *R: A Language and Environment for Statistical Computing.* Vienna: R Foundation for Statistical Computing.

[B48] Recknagel F. French M. Harkonen P. Yabunaka K.-I. (1997). Artificial neural network approach for modelling and prediction of algal blooms. *Ecol. Model.* 96 11–28. 10.1016/S0304-3800(96)00049-X

[B49] Ruiz-de la Torre M. C. Maske H. Ochoa J. Almeda-Jauregui C. O. (2013). Maintenance of coastal surface blooms by surface temperature stratification and wind drift. *PLoS One* 8:e58958. 10.1371/journal.pone.0058958 23593127 PMC3623857

[B50] Schlüter L. Møhlenberg F. Havskum H. Larsen S. (2000). The use of phytoplankton pigments for identifying and quantifying phytoplankton groups in coastal areas: testing the influence of light and nutrients on pigment/chlorophyll a ratios. *Mar. Ecol. Prog. Ser.* 192 49–63. 10.3354/meps192049

[B51] Schmitt-Jansen M. Altenburger R. (2008). Community-level microalgal toxicity assessment by multiwavelength-excitation PAM fluorometry. *Aquat. Toxicol.* 86 49–58. 10.1016/j.aquatox.2007.10.001 18036674

[B52] Schreiber U. (2004). *Pulse-Amplitude-Modulation (PAM) Fluorometry and Saturation Pulse Method: An Overview.* Würzburg: University of Wuerzburg 10.1007/978-1-4020-3218-9_11

[B53] Schreiber U. Bilger W. Neubauer C. (1995a). “Chlorophyll fluorescence as a nonintrusive indicator for rapid assessment of in vivo photosynthesis,” in *Ecophysiology of Photosynthesis*, ed. Martyn M. (Berlin: Springer), 49–70.

[B54] Schreiber U. Hormann H. Neubauer C. Klughammer C. (1995b). Assessment of photosystem II photochemical quantum yield by chlorophyll fluorescence quenching analysis. *Aust. J. Plant Physiol.* 22 209–220. 10.1071/PP9950209 20122896

[B55] Shen H. Song L. (2007). Comparative studies on physiological responses to phosphorus in two phenotypes of bloom-forming Microcystis. *Hydrobiologia* 592 475–486. 10.1007/s10750-007-0794-3

[B56] Shi P. Shen H. Wang W. Yang Q. Xie P. (2016). Habitat-specific differences in adaptation to light in freshwater diatoms. *J. Appl. Phycol.* 28 227–239. 10.1007/s10811-015-0531-7

[B57] Stumpf R. P. (2001). Applications of satellite ocean color sensors for monitoring and predicting harmful algal blooms. *Hum. Ecol. Risk Assess.* 7 1363–1368. 10.1080/20018091095050

[B58] Tao M. Xie P. Chen J. Qin B. Zhang D. Niu Y. (2012). Use of a generalized additive model to investigate key abiotic factors affecting microcystin cellular quotas in heavy bloom areas of Lake Taihu. *PLoS One* 7:e32020. 10.1371/journal.pone.0032020 22384128 PMC3285656

[B59] Tolotti M. Manca M. Angeli N. Morabito G. Thaler B. (2006). Phytoplankton and zooplankton associations in a set of Alpine high altitude lakes: geographic distribution and ecology. *Hydrobiologia* 562 99–122. 10.1007/s10750-005-1807-8

[B60] Trimbee A. M. Prepas E. (1987). Evaluation of total phosphorus as a predictor of the relative biomass of blue-green algae with emphasis on Alberta lakes. *Can. J. Fish. Aquat. Sci.* 44 1337–1342. 10.1139/f87-158

[B61] Veen G. F. Sundqvist M. K. Wardle D. A. (2015). Environmental factors and traits that drive plant litter decomposition do not determine home-field advantage effects. *Funct. Ecol.* 29 981–991. 10.1111/1365-2435.12421

[B62] Venables W. N. Dichmont C. M. (2004). GLMs, GAMs and GLMMs: an overview of theory for applications in fisheries research. *Fish. Res.* 70 319–337. 10.1016/j.fishres.2004.08.011

[B63] Watson S. B. Zastepa A. Boyer G. L. Matthews E. (2017). Algal bloom response and risk management: on-site response tools. *Toxicon* 129 144–152. 10.1016/j.toxicon.2017.02.005 28209478

[B64] Wei Z. Zhang L. Yang S. Lv X. Zhu J. Dou J. (2012). Community structure and seasonal succession of Phytoplankton in Erhai Lake. *J. Hydroecology* 1674–3075. 10.15928/j.1674-3075.2012.04.002

[B65] Wen H. Ma G. (2011). Study of water quality and algae in erhai lake during 2008–2010. *Env. Sci. Manag.* 11 44–48. 10.3969/j.issn.1673-1212.2011.11.010

[B66] White S. Anandraj A. Bux F. (2011). PAM fluorometry as a tool to assess microalgal nutrient stress and monitor cellular neutral lipids. *Bioresour. Technol.* 102 1675–1682. 10.1016/j.biortech.2010.09.097 20965719

[B67] Wickham H. (2007). Reshaping Data with the reshape Package. *J. Statist. Software* 21 1–20. 10.3978/j.issn.2305-5839.2016.01.33 27004225 PMC4779770

[B68] Wickham H. (2009). *ggplot2: Elegant Graphics for Data Analysis.* New York, NY: Springer 10.1007/978-0-387-98141-3

[B69] Winder M. Sommer U. (2012). Phytoplankton response to a changing climate. *Hydrobiologia* 698 5–16. 10.1007/s10750-012-1149-2

[B70] Wong K. T. Lee J. H. Hodgkiss I. (2007). A simple model for forecast of coastal algal blooms. *Estuar. Coast. Shelf Sci.* 74 175–196. 10.1016/j.ecss.2007.04.012

[B71] Wu Q. Wang Y. (1999). On the succession of aquatic communities in Erhai Lake. *J. Lake Sci.* 11 273–281. 10.18307/1999.0312 15852939

[B72] Wynne T. T. Stumpf R. P. Tomlinson M. C. Fahnenstiel G. L. Dyble J. (2013). Evolution of a cyanobacterial bloom forecast system in western Lake Erie: development and initial evaluation. *J. Great Lakes Res.* 39 90–99. 10.1016/j.jglr.2012.10.003

[B73] Xu F. (1996). Ecosystem health assessment of Lake Chao, a shallow eutrophic Chinese lake. *Lakes Res.* 2 101–109. 10.1016/j.jglr.2012.10.003

[B74] Zhou P. Ang B. Poh K. (2006). A trigonometric grey prediction approach to forecasting electricity demand. *Energy* 31 2839–2847. 10.1016/j.energy.2005.12.002

